# Text normalization for named entity recognition in Vietnamese tweets

**DOI:** 10.1186/s40649-016-0032-0

**Published:** 2016-12-01

**Authors:** Vu H. Nguyen, Hien T. Nguyen, Vaclav Snasel

**Affiliations:** 1 Faculty of Information Technology, Ton Duc Thang University, Ho Chi Minh City, Vietnam; 2 Faculty of Electrical Engineering and Computer Science, VSB-Technical University of Ostrava, Ostrava, Czech Republic

**Keywords:** Text normalization, Named entity recognition, Spelling error detection and correction

## Abstract

**Background:**

Named entity recognition (NER) is a task of detecting named entities in documents and categorizing them to predefined classes, such as person, location, and organization. This paper focuses on tweets posted on Twitter. Since tweets are noisy, irregular, brief, and include acronyms and spelling errors, NER in those tweets is a challenging task. Many approaches have been proposed to deal with this problem in tweets written in English, Germany, Chinese, etc., but none for Vietnamese tweets.

**Methods:**

We propose a method that normalizes a tweet before taking as an input of a learning model for NER in Vietnamese tweets. The normalization step detects spelling errors in a tweet and corrects them using an improved Dice's coefficient or n-grams. A Support Vector Machine learning algorithm is employed to learn a classifier using six different types of features.

**Results and Conclusion:**

We train our method on a training set consisting of more than 40,000 named entities and evaluate it on a testing set consisting of 3,186 named entities. The experimental results showed that our system achieves state-of-the-art performance with F1 score of 82.13%.

## Background

In recent years, social networks have become very popular. It is easy for users to share their data using online social networks. Currently, Twitter is one of the most popular social networks. According to statistics from 2011, the number of tweets was up to 140 million per day.^1^ With such a huge number of tweets being posted every day, effective extraction and processing of those data will be very beneficial, especially to information extraction applications.

Twitter provides an interactive environment that allows the users to create their own content through tweets. Since each tweet consists of only 140 characters, users tend to use acronyms, non-standard words, and social tokens. Therefore, the tweets contain many spelling errors, and this creates a significant challenge for named entity recognition (NER). Several recognition methods for named entities have been proposed for tweets in English and other languages [2, 17, 27, 31, 44]. Although there have been many approaches proposed in formal texts for NER in the Vietnamese language, none is available for Vietnamese tweets. Thus, in this paper, we propose a method for NER in Vietnamese tweets to fill the gap. The system consists of three steps, i.e., (1) normalization of tweets by detecting and correcting spelling errors; (2) capitalization classifier; and (3) recognition of named entities. Table 1 shows an example of NER according to these three steps.

**Table 1:** An example of named entity recognition

Original tweet	xe đón h$\grave{\hat{{\mathrm{o}}}}\quad$ ngọc hà gây tai nạn **kinhh** hoàng: sẽ khởi tố tài xế http://fb.me/2MwvznBbj
Step 1: Normalization	xe đón h$\grave{\hat{{\mathrm{o}}}}$ ngọc hà gây tai nạn **kinh** hoàng: sẽ khởi tố tài xế
Step 2: Capitalization	**X**e đón **H** $\grave{\hat{{\mathrm{o}}}}$ **N**gọc **H**à gây tai nạn kinh hoàng: sẽ khởi tố tài xế
Step 3: NEs recognition	Xe đón <**PER**> H$\grave{\hat{{\mathrm{o}}}}$ Ngọc Hà </**PER**> gây tai nạn kinh hoàng: sẽ khởi tố tài xế

In this paper, we present the first attempt to provide NER capability in Vietnamese tweets, and this contribution has three components, i.e., (1) a method for the normalization of Vietnamese tweets based on dictionaries and Vietnamese vocabulary structures in combination with a language model; (2) a learning model for NER in Vietnamese tweets with six different types of features; and (3) a training set of more than 40,000 named entities and a testing set of 3186 named entities to evaluate the NER system of Vietnamese tweets.

The rest of this paper is organized as follows. The second section presents earlier work related to this effort. Our proposed method is presented in third section; fourth section is the experiments and their results. Our conclusions are presented in fifth section.

## Related work

### NER

Named entity recognition has been studied extensively on formal texts, such as news and authorized web content. Several approaches have been proposed using different learning models, such as condition random fields (CRF), maximum entropy model (MEM), hidden markov model (HMM), and support vector machines (SVM). In particular, Mayfield et al. [34] used SVM to estimate lattice transition probabilities for NER. McCallum and Li [35] applied a feature induction method for CRF to recognize named entities. A combination of a CRF model and latent semantics to recognize named entities was proposed in [18]. A method using soft-constrained inference for NER was proposed in [11]. In [8] and [54], the authors proposed a maximum entropy tagger and an HMM-based chunk tagger to recognize named entities. Unfortunately, those methods gave poor performance on tweets, as pointed out in [31].

### Vietnamese NER

In the domain of Vietnamese texts, various approaches have been proposed using various learning models, such as SVM [49], classifier voting [48] and CRF [19, 52]. Some other authors have proposed other methods for NER, such as a rule-based method [36, 38], labeled propagation [21], the use of a bootstrapping algorithm and a rule-based model [51], and combined linguistically motivated and ontological features [39]. Pham et al. [41] proposed an online learning algorithm, i.e., MIRA [7] in combination with CRF and bootstrapping. Sam et al. [46] used the idea of Liao and Veeramachaneni in [28] based on CRF and expanded it by combining proper name co-references and named ambiguity heuristics with a powerful sequential learning model. Nguyen and Pham [22] proposed a feature selection approach for named entity recognition using a genetic algorithm. To calculate the accuracy of the recognition of the named entity, this paper used KNN and CRF. Nguyen and Pham [37] proposed a systematic approach to avoid the conflict between rules when a new rule was added to the set of rules for NER. Le and Tran [23] proposed some strategies to reduce the running time of genetic algorithms used in a feature selection task for NER. These strategies included reducing the size of the population during the evolution process of the genetic algorithm, reducing the fitness computation time of individuals in the genetic algorithm using progressive sampling for finding the (near) optimal sample size of the training data, and parallelization of individual fitness computation in each generation.

**Table 2:** Results of several previous works in Vietnamese NER

System	Entity types	Precision (%)	Recall (%)	F1 (%)
[19]	PER	84	82.56	83.39
[36]	PER, ORG, LOC, NA, FA, RE	92	76	83
[38]	PER, ORG, LOC	86.05	81.11	83.51
[46]	PER, ORG, LOC	93.13	88.15	79.35
[48]	PER, ORG, LOC, CUR, NUM, PERC, TIME	86.44	85.86	89.12
[49]	PER, ORG, LOC, CUR, NUM, PERC, TIME	89.05	86.49	87.75
[52]	PER, ORG, LOC, CUR, NUM, PERC, TIME, MISC	83.69	87.41	85.51

*PER* person, *ORG* organization, *LOC* location, *CUR* currency, *NUM* number, *PERC* percent, *TIME* time, *NA* nationality, *FA* facility, *RE* region, *MISC* miscellaneous

However, there have been no approaches that focused on NER in Vietnamese tweets or (short) informal Vietnamese texts.

To better collocate our results with other existing Vietnamese NER systems that used other techniques, we report the performances of other Vietnamese NER systems in Table 2.

### NER in tweets

Regarding microblog texts written in English and other languages, several approaches have been proposed for NER. Among them, Ritter et al. [44] proposed an NER system for tweets, called T-NER, which employed a CRF model for training and Labeled-LDA. Ramage et al. [43] proposed an external knowledge base, i.e., Freebase^2^ for NER. A hybrid approach to NER on tweets was presented in [31] in which a KNN-based classifier and a CRF model were used. A combination of heuristics and MEM was proposed in [17]. In [50], a semi-supervised learning approach that combined the CRF model with a classifier based on the co-occurrence coefficient of the feature words surrounding the proper noun was proposed for NER on Twitter. Li and Liu [26] proposed non-standard word (NSW) detection and decided a word is out of vocabulary (OOV) based on the dictionary, and then applied the normalization system of [25] to normalize OOV words. The results from NSW detection was used for NER based on the pipeline strategy or the joint decoding fashion method. In [32], a named entity was recognized using three steps, i.e., (1) each tweet is pre-labeled using a sequential labeler based on the linear conditional random fields (CRFs) model; (2) tweets are clustered to put those that have similar content into the same group; and (3) each cluster refines the labels of each tweet using an enhanced CRF model that incorporates the cluster-level information. Liu et al. [33] proposed jointly conducting NER and named entity normalization (NEN) for multiple tweets using a factor graph, which leverages redundancy in tweets to make up for the dearth of information in a single tweet and allows these two tasks to inform each other. Liu et al. [30] proposed a novel method for NER consisting of three core elements, i.e., normalization of tweets, combination of a KNN classifier with a linear CRF model, and a semi-supervised learning framework. Nguyen and Moschitti [40] presented a method for incorporating global features in NER using re-ranking techniques that used two kinds of features, i.e., flat and structured features and a combination of CRF and SVM. In [55], a CRF model without being focused on Gazetteers was used for NER for Arabic social media.

Recently, [1] presented the results of Shared Tasks of the 2015 Workshop on Noisy User-generated Text: Twitter Lexical Normalization and Named Entity Recognition. According to this paper, most of researchers used CRF. However, several researchers in this workshop described new methods, such as [13], which used absolutely no hand-engineered features and relied entirely on embedded words and a feed-forward, neural-network (FFNN) architecture; Cherry et al. [3] developed a semi-Markov MIRA trained tagger; Yamada et al. [53] used entity-linking-based features, and other researchers used CRFs.

Since some of the specific features of Vietnamese were presented in [49], one cannot apply those methods directly to Vietnamese tweets.

In this paper, we propose a method for NER in Vietnamese tweets to fill the gap. Our method includes three main tasks, i.e., (1) a method for normalization of Vietnamese tweets based on dictionaries and Vietnamese vocabulary structures in combination with a language model; (2) a method for detecting and correcting suitable capital letters; and (3) a model for training and recognizing named entities in Vietnamese tweets. We also conducted experiments to evaluate our NER method focused on three entity types, i.e., PERSON, LOCATION, and ORGANIZATION.

### Normalization

When we approached NER in Vietnamese tweets, we found that, on Twitter, they are noisy, irregular, brief, and consist of acronyms and spelling errors. Processing those tweets is more challenging than processing news or formal texts. To deal with this issue, several researchers have focused on other languages than Vietnamese. For example, Han et al. [14, 15] proposed a method to detect and handle errors based on the morphophonemic similarity. Choi et al. [4] detected and handled many non-standard words in online social networks using a diverse coefficient method, such as Dice, Jaccard, and Ochiai. Hassan and Menezes [16] used random walks on a contextual similarity bipartite graph constructed from* n*-gram sequences on large unlabeled text corpus to normalize social text. Sproat et al. [47] developed a novel method for normalizing and morphologically analyzing Japanese noisy text by generating both character-level and word-level normalization candidates and using discriminative methods to formulate a cost function. An approach to normalize Twitter messages in Malay based on corpus-driven analysis was proposed in [45]. Cotelo et al. [6] proposed a modular approach for lexical normalization applied to Spanish tweets. This system is proposed by including the detection of modules and candidate for correction for each out-of-vocabulary word and ranking the candidates to select the best one. Liu et al. [29] proposed a normalization system for short message service (SMS) and Twitter data based on a broad-coverage normalization system by integrating three human perspectives, i.e., enhanced letter transformation, visual priming, and string/phonetic similarity.

Recently, in the Shared Tasks of the 2015 Workshop on Noisy User-generated Text: Twitter Lexical Normalization and Named Entity Recognition, several methods were proposed for the normalization of Twitter lexical usages. According to the summary of results in [1], the common approaches were lexicon-based methods, CRF, and neural network-based methods. Among the constrained systems, neural networks achieved strong results even without off-the-shelf tools. In contrast, CRF and lexicon-based approaches were shown to be effective in the unconstrained category. Considering the Vietnamese language, we have not found any research work that has undertaken this task.

## Proposed method

**Fig. 1:**
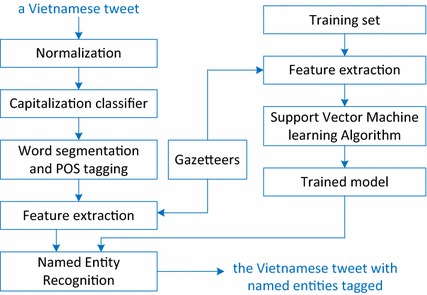
NER model for Vietnamese tweets

In this section, we present our method for NER in Vietnamese tweets. This model has two main parts, i.e., one for training and another for recognizing. Figure 1 describes our model. In our model, the gazetteers are used for both training and recognizing. We will provide more detail in the following subsections.

### The theoretical background

Currently, there are several viewpoints on what is a Vietnamese word. However, to meet the goals of automatic error detection, normalization and classification, we followed the viewpoint in [48], i.e., “A Vietnamese word is composed of special linguistic units called Vietnamese morphosyllables.” A morphosyllable may be a morpheme, a word, or something else [49]. And according to the syllable dictionary of Hoang Phe [42], we split a morphosyllable into two basic parts, consonants and syllables, as follows:

- Consonants: The Vietnamese language has 27 consonants, i.e., “b,” “ch,” “c,” “d,” “đ,” “gi,” “gh,” “g,” “h,” ’‘kh,” ’‘k,” “l,” “m,” “ngh,” “ng,” “nh,” “n,” “ph,” “q,” “r,” “s,” “th,” “tr,” “t,” “v,” “x,” “p.” In those consonants, there are eight tail consonants, i.e., “c,” “ch,” “n,” “nh,” “ng,” “m,” “p,” and “t.”
- Syllables: A syllable may be a vowel, a combination of vowels, or a combination of vowels and tail consonants. According to the syllable dictionary of Hoang Phe, the Vietnamese language has 158 syllables, and the vowels in these syllables do not occur consecutively more than once, except for the syllables “ooc” and “oong.”

Vowels: The Vietnamese language has 12 vowels, i.e., “a,” “ă,” “â,” “e,” “ê,” “i,” “o,” “ô,” “ơ,” “u,” “ư,” and “y.”

### Normalization

Because Vietnamese tweets on Twitter are noisy, irregular, and brief and consist of acronyms and spelling errors. Therefore, we propose a method to normalize them before performing NER. Our normalization method has two steps, i.e., error detection and error correction.

#### Error detection

Before performing this step, the noisy contents of tweets must be removed, such as emotion symbols (e.g., ❤❤), hashtag symbols, link url @username and others. To detect errors, we synthesized and built a dictionary for all Vietnamese morphosyllables, and it contains more than 7300 morphosyllables. A morphosyllable in a tweet will be identified as an error if it does not appear in the morphosyllable dictionary. Normally, Vietnamese tweets include two kinds of errors, i.e., typing errors and spelling errors.

#### Typing errors

Two popular typing methods are used to compose Vietnamese tweets, i.e., Telex typing and VNI typing. Each method combines letters to form Vietnamese tweets. Vietnamese characters have some extra vowels that do not exist in Latin characters, i.e., â, ă, ê, ô, ơ, one more consonant, đ; Vietnamese has five types of marks, i.e., acute accent (“á”), grave accent (“à”), hook accent (“ả”), tilde (“ã”), and heavy accent (“ạ”). The combination of vowels and marks forms the Vietnamese language its own identity.

**Example:**

- When using Telex typing, we have the combination of characters to form Vietnamese vowels, such as aa for â, aw for ă, ee for ê, oo for ô, ow for ơ, and uw for ư. Also we have one consonant, dd, for đ. For forming marks, we have s for acute accent, f for grave accent, r for hook accent, x for tilde, and j for heavy accent.
- Similar to Telex typing, we have the combination of characters in VNI typing, such as: a6 for â, a8 for ă, e6 for ê, o6 for ô, o7 for ơ, u7 for ư, and d9 for đ. To form marks, we have 1 for accent, 2 for grave accent, 3 for hook accent, 4 for tilde, and 5 for heavy accent.

Tweets are very short and prepared quickly, so the typing speed can cause errors. For example:

- With the word, “Nguyễn,” we could have typing errors such as “nguyeenx,” “nguyênx,” or “nguyeenxx” with Telex typing, and “nguye6n4,” “nguyên4,” or “nguye6n44” with VNI typing.
- With the word, “người”, we could type the following incorrect words: “ngươif,” “ngươfi,” “nguowfi,” “nguowif,” “nguofwi,” “nguofiw,” “nguoifw,” “nguoiwf,” or “nguowff” with Telex typing, and “nguwowi2,” “ngươ2i,” “nguo72i,” “nguo7i2,” “nguo27i,” “nguo2i7,” “nguoi27,” or “nguoi72” with VNI typing.

To handle this issue, we built a set of syllable rules with their tone-marks and a set of rules to map these syllables to their errors, as shown in the following examples:

- “án”: “asn,” “ans,” “a1n,” or “an1”
- “àn”: “afn,” “anf,” “a2n,” or “an2”
- “ản”: “arn,” “anr,” “a3n,” or “an3”
- “ãn”: “axn,” “anx,” “a4n,” or “an4”
- “ạn”: “ajn,” “anj,” “a5n,” or “an5”

#### Spelling errors

Spelling errors occur frequently in Vietnamese tweets. Normally, they occur due to mistakes in pronunciation. Some examples of spelling errors are as follows:

- Error due to using the wrong mark: “quyển sách” (book) to “quyễn sách”
- Initial consonant error: “bóng chuyền” (volleyball) to “bóng truyền”
- End consonant error: “bài hát” (song) to “bài hác”
- Region error: “tìm kiếm” (find) to “tìm kím”

#### Error correction

For the detected typing and spelling errors, first, the system uses vocabulary structures and the set of syllable rules to normalize them. Then, the system uses* n*-gram to normalize these results based on the degree of similarity between them.

#### a. Similarity of two morphosyllables

To measure the similarity of two morphosyllables, we used the results in the research of Dice [9] with some improvements we made. To use Dice’s research, we split all of the characters of the morphosyllables to bigrams. Assuming that we have two morphosyllables, i.e., “nguyen” and “nguye,” the bigrams of these morphosyllables can be represented as follows: bigram_nguyn_ = {ng, gu, uy, yn}, and bigram_nguyen_= {ng, gu, uy, ye,en}.

#### Dice coefficient

The Dice coefficient, developed by Lee Raymond Dice [9], is a statistical approach for comparing the similarity of two samples. The Dice coefficient of the two morphosyllables, $w_i$ and $w_j$, according to bigram can be calculated using Eq. 1:

1 $$\begin{aligned} \text {Dice}(w_i,w_j) = \frac{2 \times \mid {\text {bigram}}_{w_i}\bigcap {\text {bigram}}_{w_j} \mid }{\mid {\text {bigram}}_{w_i}\mid + \mid {\text {bigram}}_{w_j} \mid } \end{aligned}$$

where

- $\mid {\text {bigram}}_{w_i} \mid$ and $\mid {\text {bigram}}_{w_j}\mid$ are the total bigrams of $w_i$ and $w_j$
- $\mid {\text {bigram}}_{w_i}\mid \bigcap \mid {\text {bigram}}_{w_j}\mid$ are the number of bigrams which appear in $w_i$ and $w_j$ at the same time.

If two morphosyllables are the same, the Dice coefficient is 1. The higher the Dice coefficient, the higher the degree of similarity and vice versa.

#### Proposed method to improve the Dice coefficient

As observing from the experimental data using the Dice coefficient, we found that the above method will be accurate with misspelled morphosyllables which is having the misspelled character at the end. When misspelled characters occur close to the last character, at least we will lose the similarity of the last two grams. For a morphosyllable that has three characters, the degree of similarity is 0. For example: Dice(“rất”, “rát”) = 0; Dice(“gân”, “gần”) = 0;

From the above problem, we proposed a method to improve the Dice coefficient. The improvement of coefficient was performed by combining the first character with the last character of the two morphosyllables to form a new pair of bigrams. If the two members of this pair are different, the system will use the coefficients as shown in Eq. (1). In contrast, we use Eq. (2) as follows:

2 $$\begin{aligned} i{\text {Dice}}(w_i,w_j) = \frac{2 \times \left( \mid {\text {bigram}}_{w_i}\bigcap {\text {bigram}}_{w_j} \mid + \ 1 \right) }{\mid {\text {bigram}}_{w_i}\mid + \mid {\text {bigram}}_{w_j} \mid + \ 2} \end{aligned}$$

Let ${\text {fbigram}_{w}}$ be an additional bigram of w. Each fbigram is the pair of the first and the last character of w. We can express the formula for improving the Dice coefficient as Eq. (3):

3 $$\begin{aligned} f{\text {Dice}}(w_i,w_j) = \left\{ \begin{array}{l} {\text {Dice}}(w_i,w_j): \text {if fbigram}_{w_i}\;\mathrm{is \; different\; from \,fbigram}_{w_j}\\ i{\text {Dice}}(w_i,w_j): \mathrm{otherwise} \end{array} \right. \end{aligned}$$

To illustrate the improvement of the Dice coefficient, we assumed that we have two morphosyllables to measure the degree of similarity, i.e., “nguyen” and “nguyn,” as presented in the previous section, thus we have $\mid {\text {bigram}}_{w_i} \bigcap {\text {bigram}}_{w_j}\mid \,=3.$ Combining the first and the last characters of the two morphosyllables we have the new pair of bigram, which has the same result, i.e., “nn.” So, using the improvement of the Dice coefficient, we have fDice(“nguyen,” “nguyn”) = 0.727. If we use the normal coefficient of Dice, we have Dice(“nguyen,” “nguyn”) = 0.667. Table 3 shows the results of measuring the similarity of two morphosyllables with the Dice coefficient and the improved Dice coefficient methods. With the improved method, the similarities are obviously improved.

**Table 3:** The results of measuring the similarity of two morphosyllables with the Dice coefficient and the improved of Dice coefficient methods

Error morphosyllable	Correct morphosyllable	Dice	fDice
rat	rất	0	*0.333*
rat	rác	0	0
Nguễn	Nguyễn	0.667	*0.727*
Nguễn	Nguy	0.571	0.571
tượg	Tượng	0.571	*0.667*
tượg	Tương	0.286	*0.444*

#### b. Similarity of two sentences

Assume that we need to measure the similarity of two sentences, i.e., $S_1=w_1\,w_2\, \ldots w_n$ and $S_2=w'_1\,w'_2\, \ldots w'_n$. We compare the similarity of each pair of morphosyllables according to the improved Dice coefficient. Then, we compute the similarity of the two sentences by Eq. (4):

4 $$\begin{aligned} {\text {Sim}}\left( S_1,S_2\right) = \frac{\Sigma ^n_{i=1} \,f Dice \left( w_i,w'_i\right) }{n} \end{aligned}$$

where $w_i$ and $w'_i$ are the corresponding morphosyllables of $S_1$ and $S_2$.* n* is the number of morphosyllables.

If two sentences are the same, their degree of similarity (Sim) is 1. The higher the Sim coefficent, the higher the degree of similarity becomes, and vice versa. Table 4 shows the results of the normalization of Vietnamese tweets that have spelling errors.

**Table 4:** Tweets with spelling errors and their normalization

Spelling error tweets	Normalized tweets
xe đón hồ ngọc hà gây tai nạn **kinhh** hoàng: sẽ khởi tố tài xế http://fb.me/2MwvznBbj	xe đón hồ ngọc hà gây tai nạn **kinh** hoàng: sẽ khởi tố tài xế (the car picked up ho ngoc ha caused a terrible accident: the driver will be prosecuted)
hôm nay, **siinh** viên **ddaijj** học tôn **dduwcss** thắng được nghỉ học	hôm nay, **sinh** viên **đại** học tôn **đức** thắng được nghỉ học (today, students of ton duc thang university were allowed to absent)

### Capitalization classifier

Capitalization is a key orthographic feature for recognizing named entities [10, 12]. Unfortunately, in tweets, capitalization is much less reliable than edited texts. Users usually compose and reply to messages quickly, and they do not care much about capitalization. According to [5], a letter is capitalized in the following cases:

1. Capitalize the first letter of the first syllable of a complete sentence, after punctuation (.), question mark (?), exclamation point (!), ellipsis ($\ldots$) and new line.
2. Capitalize the name of people, locations, and organizations.
3. Other cases of capitalization include, e.g., medal name, position name, days of the week, months of the year, holidays, names of books, and names of magazines

Because our method focuses on three types of entities, i.e., person, organization, and location, in the capitalization classifier, we take the first and the second cases into account. For the first case, we detect the structure of the sentence and correct incorrect capitalization. In the second case, we use gazetteers of persons, locations, and organizations. Table 5 shows the results of the capitalization classifier of Vietnamese tweets.

**Table 5:** Some results of capitalization classifier of Vietnamese tweets

Tweets before capitalization	Tweets after capitalization classifier
xe đón **h** $\grave{\hat{{\mathrm{o}}}}$ **n**gọc **h**à gây tai nạn kinh hoàng: sẽ khởi tố tài xế	xe đón **H** $\grave{\hat{{\mathrm{o}}}}$ **N**gọc **H**à gây tai nạn kinh hoàng: sẽ khởi tố tài xế(the car picked up Ho Ngoc Ha caused a terrible accident: the driver will be prosecuted)
hôm nay, sinh viên đại học **t**ôn đức **t**hắng được nghı̉ học	hôm nay, sinh viên **Đ**ại học **T**ôn **Đ**ức **T**hắng được nghı̉ học (today, students of Ton Duc Thang university were allowed to absent)

### Word segmentation and part of speech (POS) tagging

To perform word segmentation and POS tagging for normalized tweets, we used vnTokenizer^3^ of [20] for word segmentation and VnTagger^4^ of [24] for POS tagging.

### Extraction of features

This phase aims to convert each word to a vector of feature values. Our system uses the IOB model to annotate data in the training and classification phases. IOB is expressed as follows:

- I: current morphosyllable is inside of a named entity (NE).
- O: current morphosyllable is outside of an NE.
- B: current morphosyllable is the beginning of an NE.

Table 6 shows the characteristic value of labels according to the IOB model with four classes, i.e., PER, LOC, ORG, and O. The selection of specific attributes from the training set has a key role in identifying the type of entity. Since the nature of the Vietnamese language is different from English, we used the most appropriate and reasonable features to achieve optimum accuracy for the system. Our system uses the following features:

- *Word position* The position of words in a sentence.
- *POS* POS tag of the current word.
- *Orthographic* Capitalization of first character, capitalization of all letters, lowercase, punctuation, numbers.
- *Gazetteer* We build several gazetteer lists, such as person, location, organization, and prefixes. These gazetteer lists consist of more than 50,000 names of people, nearly 12,000 names of locations, and 7000 names of organizations.
- *Prefix, Suffix* The first and the second character; the last and the next to the last character of the current word.
- *POS Prefix, POS Suffix* POS tags of two previous words and POS tags of two following words of the current word.

**Table 6:** The characteristic value of labels according to IOB model

Label	Value	Meaning
O	[1]	Outside a named entity
B-PER	[2]	Beginning morphosyllable of a NE belongs to a Person class
I-PER	[3]	Inside morphosyllable of a NE belongs to Person class
B-LOC	[4]	Beginning morphosyllable of a NE belongs to Location class
I-LOC	[5]	Inside morphosyllable of a NE belongs to Location class
B-ORG	[6]	Beginning morphosyllable of a NE belongs to Organization class
I-ORG	[7]	Inside morphosyllable of a NE belongs to Organization class

## Evaluation

### Data using for normalization

In this paper, to normalize for spelling errors that cannot be normalized by Vietnamese structure or a set of syllable rules, we used the tri-gram language model (tri-gram of word). This model was built from SRILM^5^ with a huge amount of data collected from online newspapers, e.g., http://www.vnexpress.net, http://nld.com.vn/, http://dantri.com.vn/, and others. The data were collected from many fields, such as current events, world, law, education, science, business, sports, and entertainment with over 429,310 articles. The total volume of collected data was about 1045 MB. The tri-gram model that was built from SRILM was about 1460 MB. To ensure the accuracy of results, we chose all of the tri-grams from the SRILM model in which the frequency of occurrences was greater than or equal five. The volume of selected tri-grams was around 81 MB, and the number of tri-grams was around 3.75 million.

### NER training set

As seen in Fig. 1, before performing feature extraction, we perform word segmentation, POS tagging, and assigning labels in Table 6 for each word in the training set. Then, the system extracts features of the words and represents each of those words as a feature vector. A support vector machine learning algorithm was used to train the model using the training set.

In particular, we assigned labels for words in the training set using a semi-automatic program, meaning that we assigned labels to those words with a program we wrote and checked in hand. In our self-written program, we considered the noun phrase obtained after the tagging step with a list of dictionary of text files to label for those words. The text files of the dictionary contain:

- The noun prefix for people such as you, sister, uncle, and president.
- The noun prefix for organizations such as company, firm, and corporation.
- The noun prefix for locations such as province, city, and district.
- List of dictionary for states, provinces of Vietnam, and others.

Table 7 shows the results of assigning labels to words of two Vietnamese tweets. The total number of entities to which we assigned labels in this phase is presented in Table 8.

**Table 7:** The results of assigning labels to words of two Vietnamese tweets

Tweets	Tweets after assigning labels
xe đón Hồ Ngọc Hà gây tai nạn kinh hoàng: sẽ khởi tố tài xế	xe đón <**PER**> Hồ Ngọc Hà </**PER**> gây tai nạn kinh hoàng: sẽ khởi tố tài xề (the car picked up Ho Ngoc Ha caused a terrible accident: the driver will be prosecuted)
hôm nay, sinh viên Đại học Tôn Đức Thắng được nghı̉ học	hôm nay, sinh viên <**ORG**> Đại học Tôn Đức Thắng </**ORG**> được nghı̉ học (today, students of Ton Duc Thang university were allowed to absent)

After assigning the labels for words in Vietnamese tweets, we analyzed these tweets to build feature vectors for those words. The structure of a feature vector includes <label> <index1>:<value1> <index2>:<value2> <index3>:<value3> and other pairs, where

- <**label**>: value from 1 to 7 according to 7 labels (O, B-PER, I-PER, B-LOC, I-LOC, B-ORG, I-ORG).
- <**index**>:<**value**>: order of feature and value corresponding to feature of a word, respectively.

After representing words in the training set as feature vectors, we used libSVM^6^ to train the model.

**Table 8:** Total number of named entities in the training set

Entity type	Number of named entities
PER	10,842
LOC	19,037
ORG	12,311

### Experiments

We conducted experiments to evaluate our method using a test set including 2,271 Vietnamese tweets and 3,186 named entities. In order to show the performance of normalization, we also conduct experiments to evaluate the proposed normalization method.

To evaluate normalization method, we ran the test on the tri-gram model with the normal Dice coefficient (Dice) and the improved Dice coefficient (fDice) to measure the similarity of the two sentences. We used three metrics to evaluate our method, i.e., the precision, the recall, and the F-Measure methods.

- Precision (P): number of correctly fixed errors divided by the total number of errors detected.
- Recall (R): number of correctly fixed errors divided by the total error.
- Balance F-measure (F1): $$F_1= \frac{2*P*R}{p+R}$$

Table 9 shows the experimental results of our normalization method. As seen in this table, the combination of our improved Dice coefficient and the tri-gram model achieved better performance than the normal Dice coefficient with the tri-gram model.

**Table 9:** The results using fDice and Dice with tri-gram model

Method	Precision (%)	Recall (%)	F-Measure (%)
Dice	83.85	82.76	83.30
fDice	89.66	88.50	89.08

To evaluate the NER method and make a comparison of the impact of the normalization of the test set, we conducted two experiments, i.e., one without normalization and capitalization classifier of tweets (Case 1) and the other with normalization and capitalization classifier of tweets (Case 2). Table 10 shows our experimental results. In this case, we also used three metrics to evaluate our method, i.e., the precision, the recall, and the Balance F-Measure.

- Precision (P): the number of correctly recognized named entities divided by the total number of named entities recognized by the NER system.
- Recall (R): the number of correctly recognized named entities divided by the total number of named entities in the test set.
- Balance F-Measure (F1): $$F_1= \frac{2*P*R}{p+R}$$

**Table 10:** Experimental results of case 1 and case 2

Case	# NEs in testing set	# recognized NEs	# correctly recognized NEs	# wrong recognized NEs	P (%)	R (%)	F1 (%)
1	3186	2593	2163	430	83.41	67.89	74.86
2	3186	2982	2533	449	84.94	79.50	82.13

According to Table 10, when we applied the normalization to the test set, the precision, recall and balance F-Measure of this test were higher than the case of the test set without normalization.

We re-implemented the state-of-the-art method proposed in [49] and compared its performance with our method. The results of this comparison are shown in Table 11.

**Table 11:** Comparison performance of our method with that of [49]

System	Precision (%)	Recall (%)	F1 (%)
Our system	84.94	79.50	82.13
System of [49]	83.10	77.62	80.27

## Conclusions

In this paper, we present the first attempt to NER in Vietnamese tweets on Twitter. We proposed a method for the normalization of Vietnamese tweets, based on the dictionaries and Vietnamese vocabulary structures in combination with a language model. We also proposed a learning model to recognize named entities using six different types of features. To evaluate for our normalization method, we built a tri-gram model that had a volume of about 81 MB and the number of tri-grams was around 3.75 million. The improvement in measuring the similarity of two words based on the modified Dice coefficient outperformed the original Dice coefficient, and our normalization method achieved a high performance with F1 score of 89.08%. To evaluate the NER method, we built a training set of more than 40,000 named entities and a testing set of 3186 named entities to evaluate our system. The experimental results showed that our system achieved encouraging performance, with 82.13% F1 score.

We plan to acquire a larger dataset to build and test the language model with bigram, trigram, and four-gram to improve our normalization performance. In addition, we also collected the data required to increase the number of named entities in the training set as well as to expand the Gazetteers so that we can increase the NER performance of our system.

## Footnotes

1
https://blog.twitter.com/2011/numbers.

2
http://www.freebase.com.

3
http://mim.hus.vnu.edu.vn/phuonglh/softwares/vnTokenizer.

4
http://mim.hus.vnu.edu.vn/phuonglh/softwares/vnTagger.

5
http://www.speech.sri.com/projects/srilm/.

6
http://www.csie.ntu.edu.tw/cjlin/libsvm/#download.
